# Aqua­{6,6′-dimeth­oxy-2,2′-[ethane-1,2-diylbis(nitrilo­methyl­idyne)]diphenolato}nickel(II)

**DOI:** 10.1107/S1600536809034278

**Published:** 2009-09-05

**Authors:** Zhenghua Guo, Lianzhi Li, Tao Xu, Jinghong Li, Daqi Wang

**Affiliations:** a School of Chemistry and Chemical Engineering, Liaocheng University, Shandong 252059, People’s Republic of China

## Abstract

The title complex, [Ni(C_18_H_18_N_2_O_4_)(H_2_O)], lies on a mirror plane with the Ni^II^ ion coordinated by two N and two O atoms of a tetra­dentate Schiff base ligand and one water O atom in a distorted square-pyramidal enviroment. The –CH_2_–CH_2_– group of the ligand is disordered equally over two sites about the mirror plane. The dihedral angle between the mean planes of the two symmetry-related chelate rings is 37.16 (6)°. In the crystal structure, inter­molecular O—H⋯O hydrogen bonds link complex mol­ecules into one-dimensional chains along [100] and these chains are linked, in turn, by very weak inter­molecular C—H⋯O hydrogen bonds into a two-dimensional network.

## Related literature

For background to Schiff base complexes, see: Akine *et al.* (2005 ▶); Gamovski *et al.* (1993 ▶); Garg & Kumar (2003 ▶); Tarafder *et al.* (2002 ▶); Yang *et al.* (2000 ▶). For a related crystal structure, see: Wang *et al.* (2007 ▶).
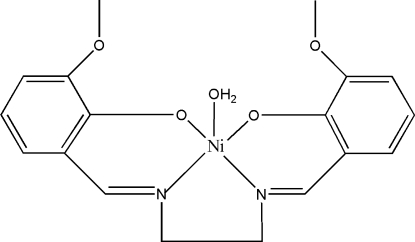


## Experimental

### 

#### Crystal data

[Ni(C_18_H_18_N_2_O_4_)(H_2_O)]
*M* *_r_* = 403.07
Orthorhombic, 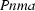

*a* = 9.2712 (11) Å
*b* = 24.763 (3) Å
*c* = 7.5185 (10) Å
*V* = 1726.1 (4) Å^3^
*Z* = 4
Mo *K*α radiation
μ = 1.16 mm^−1^
*T* = 298 K
0.48 × 0.42 × 0.26 mm

#### Data collection

Bruker SMART 1000 CCD area-detector diffractometer
Absorption correction: multi-scan (*SADABS*; Sheldrick, 1996 ▶) *T*_min_ = 0.607, *T*_max_ = 0.753
7520 measured reflections
1550 independent reflections
1368 reflections with *I* > 2σ(*I*)
*R*_int_ = 0.029

#### Refinement

*R*[*F* ^2^ > 2σ(*F* ^2^)] = 0.031
*wR*(*F* ^2^) = 0.078
*S* = 1.19
1550 reflections
131 parameters
H-atom parameters constrained
Δρ_max_ = 0.16 e Å^−3^
Δρ_min_ = −0.53 e Å^−3^

### 

Data collection: *SMART* (Siemens, 1996 ▶); cell refinement: *SAINT* (Siemens, 1996 ▶); data reduction: *SAINT*; program(s) used to solve structure: *SHELXS97* (Sheldrick, 2008 ▶); program(s) used to refine structure: *SHELXL97* (Sheldrick, 2008 ▶); molecular graphics: *SHELXTL* (Sheldrick, 2008 ▶); software used to prepare material for publication: *SHELXTL*.

## Supplementary Material

Crystal structure: contains datablocks global, I. DOI: 10.1107/S1600536809034278/lh2877sup1.cif

Structure factors: contains datablocks I. DOI: 10.1107/S1600536809034278/lh2877Isup2.hkl

Additional supplementary materials:  crystallographic information; 3D view; checkCIF report

## Figures and Tables

**Table:** 

Ni1—O1	1.9364 (16)
Ni1—N1	1.956 (2)
Ni1—O3	2.363 (2)

**Table:** 

O1—Ni1—O1^i^	90.74 (10)
O1—Ni1—N1^i^	167.34 (9)
O1—Ni1—N1	92.11 (8)
N1^i^—Ni1—N1	82.55 (14)
O1—Ni1—O3	97.90 (7)
N1—Ni1—O3	93.93 (9)

Symmetry code: (i) 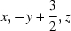.

**Table 2:** Hydrogen-bond geometry (Å, °)

*D*—H⋯*A*	*D*—H	H⋯*A*	*D*⋯*A*	*D*—H⋯*A*
O3—H3⋯O1^ii^	0.85	2.29	3.007 (3)	142
O3—H3⋯O2^ii^	0.85	2.18	2.9313 (19)	147
C10—H10*B*⋯O1^iii^	0.97	2.53	3.236 (7)	130
C9—H9*B*⋯O3^ii^	0.97	2.66	3.322 (7)	126

Symmetry codes: (ii) 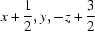; (iii) 
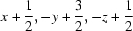
.

**Fig. 1.**
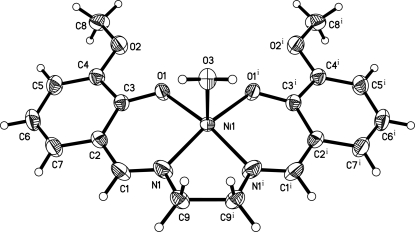
The molecular structure of the title compound, showing 30% probability displacement ellipsoids and the atom-numbering scheme. The disorder is not shown [symmetry code: (i) x, -y+3/2, z].

**Fig. 2.**
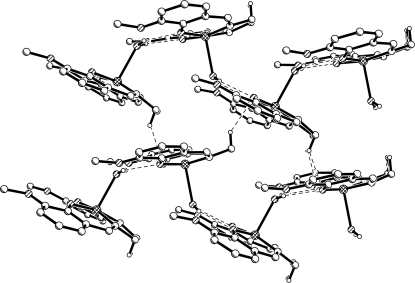
Part of the crystal structure with hydrogen bonds shown as dashed lines. Only H atoms involved in hydrogen bonds are shown.

## supplementary crystallographic information

### Comment

Schiff base complexes play an important role in the stereochemical models of transition metal coordination chemistry with their easy preparation, diversition and structural variation (Gamovski *et al.*,1993). They also have been intensively investigated owing to their strong coordination capability and diverse biological activities, such as antibacterial and antitumor activities (Yang *et al.*, 2000; Tarafder *et al.*, 2002). Therefore, synthesis of new shiff base Nickel(II) complexes is still the aim of many recent investigations (Garg & Kumar, 2003; Akine *et al.*, 2005). As part of a series of crystal structure studies (Wang *et al.*, 2007), we report here the synthesis and crystal structure of the title compound.

In the molecular structure (Fig. 1), The Ni^II^ ion is five coordinated by two N and two O atoms of a new tetradentate Schiff base ligand and one O atom of water molecule in a distorted square-pyramidal configuration. Two nitrogen atoms and two oxygen atoms of Schiff base occupy the basal plane, and the O atom of the coordinated water molecule is in the apical position. The dihedral angle between the planes of the two symmetry realted Ni/N/C/C/C/O chelate rings is 37.16 (6)°. The molecule lies on a mirror plane and the -CH_2_-CH_2_- group of the ligand is disordered equally over two sites about the mirror plane.

In the crystal structure, intermolecular O—H···O hydrogen bonds link complex molecules into one-dimensional chains along [100] and these chains are linked, in turn, by very weak intermolecular C—H···O hydrogen bonds into a two-dimensional network (Fig. 2).

### Experimental

1,2-ethylenediamine (1 mmol, 60.10 mg) was dissolved in hot methanol (10 ml) and added dropwise to a methanol solution (3 ml) of 3-methoxysalicylaldehyde (1 mmol, 152.14 mg). The mixture was then stirred at 323 K for 2 h. Subsequently, an aqueous solution (2 ml) of nickel chloride (1 mmol, 237.69 mg) was added dropwise and stirred for another 5 h. The solution was left at room temperature for 15 days, whereupon green block crystals suitable for X-ray diffraction were obtained.

### Refinement

All H atoms were placed in geometrically calculated positions (C—H = 0.93–0.97 Å, O—H = 0.85 Å) and allowed to ride on their respective parent atoms, with *U*_iso_(H) = 1.2*U*_eq_(C), 1.5*U*_eq_(methyl C) or 1.2*U*_eq_(O).

### Crystal data

**Table:**

[Ni(C_18_H_18_N_2_O_4_)(H_2_O)]	*F*(000) = 840
*M**_r_* = 403.07	*D*_x_ = 1.551 Mg m^−^^3^
Orthorhombic, *P**n**m**a*	Mo *K*α radiation, λ = 0.71073 Å
Hall symbol: -P 2ac 2n	Cell parameters from 3742 reflections
*a* = 9.2712 (11) Å	θ = 2.5–27.9°
*b* = 24.763 (3) Å	µ = 1.16 mm^−^^1^
*c* = 7.5185 (10) Å	*T* = 298 K
*V* = 1726.1 (4) Å^3^	Block, green
*Z* = 4	0.48 × 0.42 × 0.26 mm

### Data collection

**Table:**

Bruker SMART 1000 CCD area-detector diffractometer	1550 independent reflections
Radiation source: fine-focus sealed tube	1368 reflections with *I* > 2σ(*I*)
graphite	*R*_int_ = 0.029
φ and ω scans	θ_max_ = 25.0°, θ_min_ = 1.6°
Absorption correction: multi-scan (*SADABS*; Sheldrick, 1996)	*h* = −11→11
*T*_min_ = 0.607, *T*_max_ = 0.753	*k* = −29→27
7520 measured reflections	*l* = −5→8

### Refinement

**Table:**

Refinement on *F*^2^	Primary atom site location: structure-invariant direct methods
Least-squares matrix: full	Secondary atom site location: difference Fourier map
*R*[*F*^2^ > 2σ(*F*^2^)] = 0.031	Hydrogen site location: inferred from neighbouring sites
*wR*(*F*^2^) = 0.078	H-atom parameters constrained
*S* = 1.19	*w* = 1/[σ^2^(*F*_o_^2^) + (0.0321*P*)^2^ + 0.8008*P*] where *P* = (*F*_o_^2^ + 2*F*_c_^2^)/3
1550 reflections	(Δ/σ)_max_ = 0.001
131 parameters	Δρ_max_ = 0.16 e Å^−^^3^
0 restraints	Δρ_min_ = −0.53 e Å^−^^3^

### Special details

**Table:**

Geometry. All e.s.d.'s (except the e.s.d. in the dihedral angle between two l.s. planes) are estimated using the full covariance matrix. The cell e.s.d.'s are taken into account individually in the estimation of e.s.d.'s in distances, angles and torsion angles; correlations between e.s.d.'s in cell parameters are only used when they are defined by crystal symmetry. An approximate (isotropic) treatment of cell e.s.d.'s is used for estimating e.s.d.'s involving l.s. planes.
Refinement. Refinement of *F*^2^ against ALL reflections. The weighted *R*-factor *wR* and goodness of fit *S* are based on *F*^2^, conventional *R*-factors *R* are based on *F*, with *F* set to zero for negative *F*^2^. The threshold expression of *F*^2^ > σ(*F*^2^) is used only for calculating *R*-factors(gt) *etc*. and is not relevant to the choice of reflections for refinement. *R*-factors based on *F*^2^ are statistically about twice as large as those based on *F*, and *R*- factors based on ALL data will be even larger.

### Fractional atomic coordinates and isotropic or equivalent isotropic displacement parameters (Å^2^)

**Table:**

	*x*	*y*	*z*	*U*_iso_*/*U*_eq_	Occ. (<1)
Ni1	0.42518 (4)	0.7500	0.52345 (5)	0.03327 (16)	
N1	0.5687 (2)	0.69791 (9)	0.4405 (3)	0.0577 (6)	
O1	0.28010 (18)	0.69435 (6)	0.5505 (2)	0.0442 (4)	
O2	0.04869 (19)	0.63591 (7)	0.5874 (3)	0.0602 (5)	
O3	0.5119 (2)	0.7500	0.8191 (3)	0.0477 (6)	
H3	0.5568	0.7224	0.8569	0.057*	
C1	0.5493 (3)	0.64757 (11)	0.4103 (4)	0.0545 (7)	
H1	0.6273	0.6282	0.3657	0.065*	
C2	0.4180 (3)	0.61846 (10)	0.4391 (3)	0.0451 (6)	
C3	0.2919 (3)	0.64343 (9)	0.5057 (3)	0.0402 (6)	
C4	0.1686 (3)	0.60950 (10)	0.5265 (3)	0.0460 (6)	
C5	0.1735 (3)	0.55512 (11)	0.4884 (4)	0.0587 (8)	
H5	0.0919	0.5339	0.5055	0.070*	
C6	0.2999 (4)	0.53165 (11)	0.4244 (4)	0.0675 (9)	
H6	0.3025	0.4950	0.3984	0.081*	
C7	0.4188 (3)	0.56250 (11)	0.4003 (4)	0.0592 (7)	
H7	0.5028	0.5466	0.3574	0.071*	
C8	−0.0821 (3)	0.60639 (13)	0.5993 (4)	0.0647 (8)	
H8A	−0.1042	0.5908	0.4855	0.097*	
H8B	−0.1587	0.6302	0.6344	0.097*	
H8C	−0.0719	0.5782	0.6859	0.097*	
C9	0.7178 (7)	0.7189 (3)	0.4502 (9)	0.0473 (14)	0.50
H9A	0.7867	0.6957	0.3901	0.057*	0.50
H9B	0.7479	0.7246	0.5723	0.057*	0.50
C10	0.6943 (7)	0.7720 (3)	0.3518 (9)	0.0544 (17)	0.50
H10A	0.7810	0.7939	0.3574	0.065*	0.50
H10B	0.6726	0.7650	0.2278	0.065*	0.50

### Atomic displacement parameters (Å^2^)

**Table:**

	*U*^11^	*U*^22^	*U*^33^	*U*^12^	*U*^13^	*U*^23^
Ni1	0.0290 (2)	0.0330 (2)	0.0378 (3)	0.000	0.00299 (18)	0.000
N1	0.0405 (12)	0.0549 (14)	0.0778 (16)	−0.0030 (10)	0.0128 (12)	−0.0225 (12)
O1	0.0373 (9)	0.0355 (9)	0.0597 (11)	−0.0010 (7)	0.0046 (8)	−0.0087 (8)
O2	0.0454 (11)	0.0488 (11)	0.0864 (14)	−0.0098 (9)	0.0128 (10)	−0.0104 (10)
O3	0.0477 (14)	0.0425 (13)	0.0530 (15)	0.000	−0.0102 (12)	0.000
C1	0.0427 (15)	0.0547 (17)	0.0660 (18)	0.0056 (13)	0.0070 (13)	−0.0214 (14)
C2	0.0498 (15)	0.0426 (14)	0.0428 (14)	0.0031 (12)	0.0001 (12)	−0.0074 (11)
C3	0.0437 (14)	0.0385 (13)	0.0386 (13)	0.0008 (11)	−0.0034 (11)	−0.0022 (10)
C4	0.0468 (15)	0.0422 (14)	0.0492 (15)	−0.0034 (11)	0.0006 (12)	−0.0051 (11)
C5	0.0609 (18)	0.0424 (15)	0.073 (2)	−0.0111 (13)	0.0024 (15)	−0.0039 (13)
C6	0.081 (2)	0.0335 (14)	0.088 (2)	0.0001 (15)	0.0074 (19)	−0.0114 (14)
C7	0.0620 (18)	0.0454 (15)	0.0703 (19)	0.0081 (14)	0.0064 (15)	−0.0127 (14)
C8	0.0517 (17)	0.075 (2)	0.0676 (19)	−0.0234 (15)	0.0137 (15)	−0.0146 (16)
C9	0.035 (3)	0.051 (3)	0.056 (4)	0.005 (2)	0.000 (3)	−0.012 (3)
C10	0.036 (3)	0.062 (4)	0.065 (4)	−0.001 (3)	0.011 (3)	0.010 (3)

### Geometric parameters (Å, °)

**Table:**

Ni1—O1	1.9364 (16)	C5—C6	1.393 (4)
Ni1—O1^i^	1.9364 (16)	C5—H5	0.9300
Ni1—N1^i^	1.956 (2)	C6—C7	1.353 (4)
Ni1—N1	1.956 (2)	C6—H6	0.9300
Ni1—O3	2.363 (2)	C7—H7	0.9300
N1—C1	1.280 (3)	C8—H8A	0.9600
N1—C9	1.479 (7)	C8—H8B	0.9600
N1—C10^i^	1.535 (7)	C8—H8C	0.9600
O1—C3	1.310 (3)	C9—C10^i^	0.803 (7)
O2—C4	1.369 (3)	C9—C10	1.525 (7)
O2—C8	1.419 (3)	C9—C9^i^	1.541 (13)
O3—H3	0.8501	C9—H9A	0.9700
C1—C2	1.431 (4)	C9—H9B	0.9700
C1—H1	0.9300	C10—C9^i^	0.803 (7)
C2—C3	1.414 (3)	C10—C10^i^	1.092 (13)
C2—C7	1.416 (4)	C10—N1^i^	1.535 (7)
C3—C4	1.428 (3)	C10—H10A	0.9700
C4—C5	1.377 (4)	C10—H10B	0.9700
			
O1—Ni1—O1^i^	90.74 (10)	C6—C7—H7	119.3
O1—Ni1—N1^i^	167.34 (9)	C2—C7—H7	119.3
O1^i^—Ni1—N1^i^	92.11 (8)	O2—C8—H8A	109.5
O1—Ni1—N1	92.11 (8)	O2—C8—H8B	109.5
O1^i^—Ni1—N1	167.34 (9)	H8A—C8—H8B	109.5
N1^i^—Ni1—N1	82.55 (14)	O2—C8—H8C	109.5
O1—Ni1—O3	97.90 (7)	H8A—C8—H8C	109.5
O1^i^—Ni1—O3	97.90 (7)	H8B—C8—H8C	109.5
N1^i^—Ni1—O3	93.93 (9)	C10^i^—C9—N1	78.4 (8)
N1—Ni1—O3	93.93 (9)	C10^i^—C9—C10	43.4 (8)
C1—N1—C9	118.9 (3)	N1—C9—C10	98.4 (5)
C1—N1—C10^i^	120.1 (3)	C10^i^—C9—C9^i^	73.8 (8)
C9—N1—C10^i^	30.8 (3)	N1—C9—C9^i^	110.6 (3)
C1—N1—Ni1	127.10 (19)	C10—C9—C9^i^	30.4 (3)
C9—N1—Ni1	112.9 (3)	C10^i^—C9—H9A	85.1
C10^i^—N1—Ni1	109.6 (3)	N1—C9—H9A	112.6
C3—O1—Ni1	126.86 (15)	C10—C9—H9A	112.2
C4—O2—C8	118.0 (2)	C9^i^—C9—H9A	126.2
Ni1—O3—H3	118.8	C10^i^—C9—H9B	155.4
N1—C1—C2	125.7 (2)	N1—C9—H9B	111.5
N1—C1—H1	117.2	C10—C9—H9B	112.0
C2—C1—H1	117.2	C9^i^—C9—H9B	81.6
C3—C2—C7	120.3 (2)	H9A—C9—H9B	109.8
C3—C2—C1	122.4 (2)	C9^i^—C10—C10^i^	106.2 (8)
C7—C2—C1	117.2 (2)	C9^i^—C10—C9	75.9 (9)
O1—C3—C2	125.5 (2)	C10^i^—C10—C9	30.4 (3)
O1—C3—C4	118.1 (2)	C9^i^—C10—N1^i^	70.7 (8)
C2—C3—C4	116.3 (2)	C10^i^—C10—N1^i^	119.0 (3)
O2—C4—C5	124.3 (2)	C9—C10—N1^i^	108.4 (5)
O2—C4—C3	113.9 (2)	C9^i^—C10—H10A	65.1
C5—C4—C3	121.7 (3)	C10^i^—C10—H10A	124.0
C4—C5—C6	120.5 (3)	C9—C10—H10A	110.1
C4—C5—H5	119.8	N1^i^—C10—H10A	109.8
C6—C5—H5	119.8	C9^i^—C10—H10B	172.9
C7—C6—C5	119.7 (3)	C10^i^—C10—H10B	79.6
C7—C6—H6	120.1	C9—C10—H10B	109.9
C5—C6—H6	120.1	N1^i^—C10—H10B	110.4
C6—C7—C2	121.4 (3)	H10A—C10—H10B	108.4
			
O1—Ni1—N1—C1	4.5 (3)	C8—O2—C4—C5	−5.1 (4)
O1^i^—Ni1—N1—C1	−98.3 (4)	C8—O2—C4—C3	175.3 (2)
N1^i^—Ni1—N1—C1	−163.9 (2)	O1—C3—C4—O2	2.0 (3)
O3—Ni1—N1—C1	102.6 (3)	C2—C3—C4—O2	−178.5 (2)
O1—Ni1—N1—C9	−162.9 (3)	O1—C3—C4—C5	−177.7 (2)
O1^i^—Ni1—N1—C9	94.2 (5)	C2—C3—C4—C5	1.8 (4)
N1^i^—Ni1—N1—C9	28.6 (4)	O2—C4—C5—C6	179.1 (3)
O3—Ni1—N1—C9	−64.8 (3)	C3—C4—C5—C6	−1.3 (4)
O1—Ni1—N1—C10^i^	164.1 (3)	C4—C5—C6—C7	0.3 (5)
O1^i^—Ni1—N1—C10^i^	61.3 (5)	C5—C6—C7—C2	0.1 (5)
N1^i^—Ni1—N1—C10^i^	−4.3 (3)	C3—C2—C7—C6	0.5 (5)
O3—Ni1—N1—C10^i^	−97.8 (3)	C1—C2—C7—C6	179.6 (3)
O1^i^—Ni1—O1—C3	162.41 (15)	C1—N1—C9—C10^i^	101.3 (8)
N1^i^—Ni1—O1—C3	59.4 (4)	Ni1—N1—C9—C10^i^	−90.2 (8)
N1—Ni1—O1—C3	−5.2 (2)	C1—N1—C9—C10	140.0 (4)
O3—Ni1—O1—C3	−99.51 (19)	C10^i^—N1—C9—C10	38.8 (7)
C9—N1—C1—C2	163.8 (4)	Ni1—N1—C9—C10	−51.4 (4)
C10^i^—N1—C1—C2	−160.6 (4)	C1—N1—C9—C9^i^	168.8 (2)
Ni1—N1—C1—C2	−2.9 (5)	C10^i^—N1—C9—C9^i^	67.6 (8)
N1—C1—C2—C3	0.2 (5)	Ni1—N1—C9—C9^i^	−22.6 (3)
N1—C1—C2—C7	−178.9 (3)	C10^i^—C9—C10—C9^i^	180.000 (4)
Ni1—O1—C3—C2	4.5 (3)	N1—C9—C10—C9^i^	116.8 (6)
Ni1—O1—C3—C4	−176.08 (17)	N1—C9—C10—C10^i^	−63.2 (6)
C7—C2—C3—O1	178.0 (3)	C9^i^—C9—C10—C10^i^	180.000 (10)
C1—C2—C3—O1	−1.0 (4)	C10^i^—C9—C10—N1^i^	116.3 (6)
C7—C2—C3—C4	−1.4 (4)	N1—C9—C10—N1^i^	53.1 (4)
C1—C2—C3—C4	179.5 (2)	C9^i^—C9—C10—N1^i^	−63.7 (6)

Symmetry codes: (i) *x*, −*y*+3/2, *z*.

### Hydrogen-bond geometry (Å, °)

**Table:**

*D*—H···*A*	*D*—H	H···*A*	*D*···*A*	*D*—H···*A*
O3—H3···O1^ii^	0.85	2.29	3.007 (3)	142
O3—H3···O2^ii^	0.85	2.18	2.9313 (19)	147
C10—H10B···O1^iii^	0.97	2.53	3.236 (7)	130
C9—H9B···O3^ii^	0.97	2.66	3.322 (7)	126

Symmetry codes: (ii) *x*+1/2, *y*, −*z*+3/2; (iii) *x*+1/2, −*y*+3/2, −*z*+1/2.
